# Reduced exercise capacity in pediatric post-COVID syndrome correlates with time post infection and does not affect quality of life

**DOI:** 10.3389/fped.2025.1567974

**Published:** 2025-09-08

**Authors:** Lothar Stein, Svea Mackenrodt, Momme Kück, Sven Haufe, Arno Kerling, Uwe Tegtbur, Valentina Skerries, Martin Wetzke, Christine Happle

**Affiliations:** ^1^ Department of Rehabilitation and Sports Medicine, Hannover Medical School, Hannover, Germany; ^2^ Department of Pediatric Pulmology, Allergology and Neonatology, Hannover Medical School, Hannover, Germany; ^3^ Biomedical Research in Endstage and Obstructive Lung Disease (BREATH), Member of the German Center for Lung Research (DZL), Hannover, Germany; ^4^ Excellence Cluster Resolving Infection Susceptibility RESIST (EXC 2155), Deutsche Forschungsgemeinschaft, Hannover Medical School, Hannover, Germany

**Keywords:** post-COVID syndrome, pediatric, exercise testing, quality of life, SARS-CoV-2

## Abstract

**Objectives:**

Post-COVID syndrome (PCS) in children and adolescents is reported less frequently and typically of shorter duration than in adults but can be associated with significant morbidity and reduction of quality of life (QoL). In pediatric PCS, data on exercise capacity (EC) are rare.

**Methods:**

This prospective, monocentric, cross-sectional study, analyzed EC, QoL, and clinical parameters in pediatric patients with PCS (*n* = 29/53 girls, 14.4 ± 2.5 years). A total of 210.0 ± 104.2 days passed between SARS-CoV-2 infection and study-related examinations.

**Results:**

The main PCS symptoms were reduced subjective EC (92.4%), shortness of breath (64.2%), concentration difficulties (60.4%), and breathlessness (47.2%). In patients with PCS, EC was 76.6 ± 16.0% VO_2peak_predicted and maximum workload 76.0 ± 17.9% norm. Overall QoL (Kindl-R total score) was 89.2 ± 17.3% norm, self-assessed physical wellbeing was 60.7 ± 30.4% norm, and emotional wellbeing was 85.1 ± 23.2% norm. We grouped the PCS patients into deconditioned vs. conditioned (threshold 80% of VO_2peak_predicted EC). No group differences in age, bodyweight, height, muscle mass, fat percentage, BMI, lung function, neuropsychological symptoms, and wellbeing were found. While maximum workloads and VO_2peak_ differed significantly according to grouping, lactate levels and self-assessed exertion were comparable. However, elapsed time after SARS-COV-2 infection was significantly shorter in deconditioned vs. conditioned patients (mean 198.5 ± 120.7 vs. 230.8 ± 62.6 days; *p* = 0.021).

**Conclusion:**

Pediatric PCS is associated with reduced EC, which is significantly impacted by time post SARS-CoV-2 infection, but does not appear to affect the QoL or self-esteem of the patients in this study.

## Introduction

Post-COVID syndrome (PCS) may be diagnosed when physical, psychological, and cognitive complaints persist, recur, or fluctuate >4 weeks after acute infection with SARS-CoV-2 and when alternative etiologies are excluded (1). In children and adolescents, PCS-typical complaints are rare and typically of shorter duration than in adults (2). Nevertheless, pediatric PCS is associated with significant morbidity and a reduction in the quality of life (QoL) (3). In adults, a particular feature of PCS complaints associated with reduced QoL is exercise intolerance associated with poor lung function (4). Hemodynamic and gas exchange derangements show substantial overlaps in myalgic encephalitis/chronic fatigue syndrome (ME/CFS) and PCS, suggestive of shared mechanisms between these diseases (5). In pediatric PCS, however, data on exercise intolerance are rare. It is estimated that around 12% of children with PCS display prolonged reduction in exercise tolerance (6), but systematic data on exercise performance and how this relates to QoL in pediatric PCS are scarce.

## Methods

Baseline data of exercise capacity (EC) and related factors as well as QoL from a prospective, monocentric, cross-sectional study, in pediatric patients with PCS (*n* = 53) were analyzed. Between May 2022 and October 2023, patients referred to the pediatric PCS outpatient clinic at Hannover Medical School underwent exercise testing in the PCS-LoCoKi-study (German Clinical Trials Register: DRKS00028963). Written informed consent was obtained from all participants (ethics approval Hannover Medical School No. 9822_BO-S_2021). All patients were between 8 and 18 years old with PCS-typical complaints for at least 16 weeks post PCR-confirmed SARS-CoV-2 infection. Exclusion of pathologies other than PCS underlying PCS-typical complaints was performed through an extensive workup including thorough laboratory testing, echocardiography, electrocardiogram, lung function testing, chest x-ray, and abdominal ultrasound. Body fat was assessed by a bioimpedance analysis (InBody 720, Biospace, Seoul, Korea). Cardiopulmonary exercise testing (CPET) was performed employing a spirometric system (Oxycon CPX, CareFusion, Würzburg, Germany) on a speed-independent bicycle ergometer (Ergoline P150, Bitz, Germany) under ECG surveillance with 60–70 rpm. Incremental CPETs were performed according to the Godfrey protocol, and subjective exertion was assessed by the Borg scaling method (7). Heart rate and oxygen uptake/vital capacity (VC) were continuously monitored, and lactate was extracted from the earlobe. Bell's Functionality Score was used for a self-assessment of disability and fatigue, and fatigue was further estimated with the Fatigue Severity Scale (FSS) (8). To prevent postexertional malaise, exercise testing was adapted to the patient's performance, according to WHO recommendations (9). For health-related QoL, the pediatric Kindl-R was used (10). Data were examined for normal distribution and variance homogeneity using the Shapiro–Wilk and Levene tests. Chi-square tests were performed on frequency distributions. To determine differences between groups, either a two-tailed Student's t-test or the Mann–Whitney test was performed, with Hedges’ g effect size used to quantify the magnitude of the effect. Effect sizes ranging from 0.2 to 0.5 were classified as small, those ranging from 0.5 to 0.8 were classified as medium, and those exceeding 0.8 were classified as large. The type I error was set at 5% (two-sided). Statistical analyses were performed using SPSS (V29, IBM Corp., Armonk, NY, USA).

## Results

The mean age of the patients was 14.4 ± 2.5 years; *n* = 24/53 were boys and *n* = 29/53 were girls. A mean of 210.0 ± 104.2 days had passed between SARS-CoV-2 infection and study-related examinations. The most prevalent PCS complaints were reduced subjective EC (92.4%), shortness of breath (64.2%), and concentration difficulties (60.4%), followed by breathlessness (47.2%), sleeping disorders (37.4%), muscle pain (34.0%), headache (34.0%), dizziness (32.0%), chest pain (30.2%), and fatigue (28.3%). When comparing the overall EC from our cohort with age-and sex-matched normal values, the VO_2peak_ predicted was reduced to 76.6 ± 16.0% norm, and the maximum workload was reduced to 76.0 ± 17.9% norm. High levels of perceived exertion (Borg 17.6 ± 1.6) and respiratory exchange ratios (109.7 ± 10.9% norm) as well as a maximal lactate concentration of 6.1 ± 2.4 mmol indicated that the participants were clearly engaged in exercise testing and reached high levels of individual skeletal muscle exhaustion. No significant sex-specific differences were found for EC (78.1 ± 13.5% in girls and 75.6 ± 18.9% in boys; *p* = 0.90), for VO_2peak_ (77.9 ± 18.0% in girls and 73.7 ± 17.8% in boys; *p* = 0.44), or for any further exertion values.

With regard to QoL, pediatric patients with PCS reported slightly lower levels than normal. Across all participants, the Kindl-R total score representing the estimation of overall QoL was 89.2 ± 17.3% of normal. Self-assessed physical wellbeing (60.7 ± 30.4% of norm) appeared to be more strongly affected by pediatric PCS than emotional wellbeing (85.1 ± 23.2% of norm). Also for these values, no sex-specific differences were observed (QoL in girls 92.2 ± 19.9% vs. boys 85.6 ± 13.2%; *p* *=* 0.12; self-assessed physical wellbeing in girls 63.3 ± 34.4% vs. boys 57.7 ± 25.2%; *p*-value 0.50, self-assessed emotional wellbeing in girls 87.3 ± 27.2% and in boys 85.6 ± 17.7%; *p* *=* 0.35).

**Figure 1:**
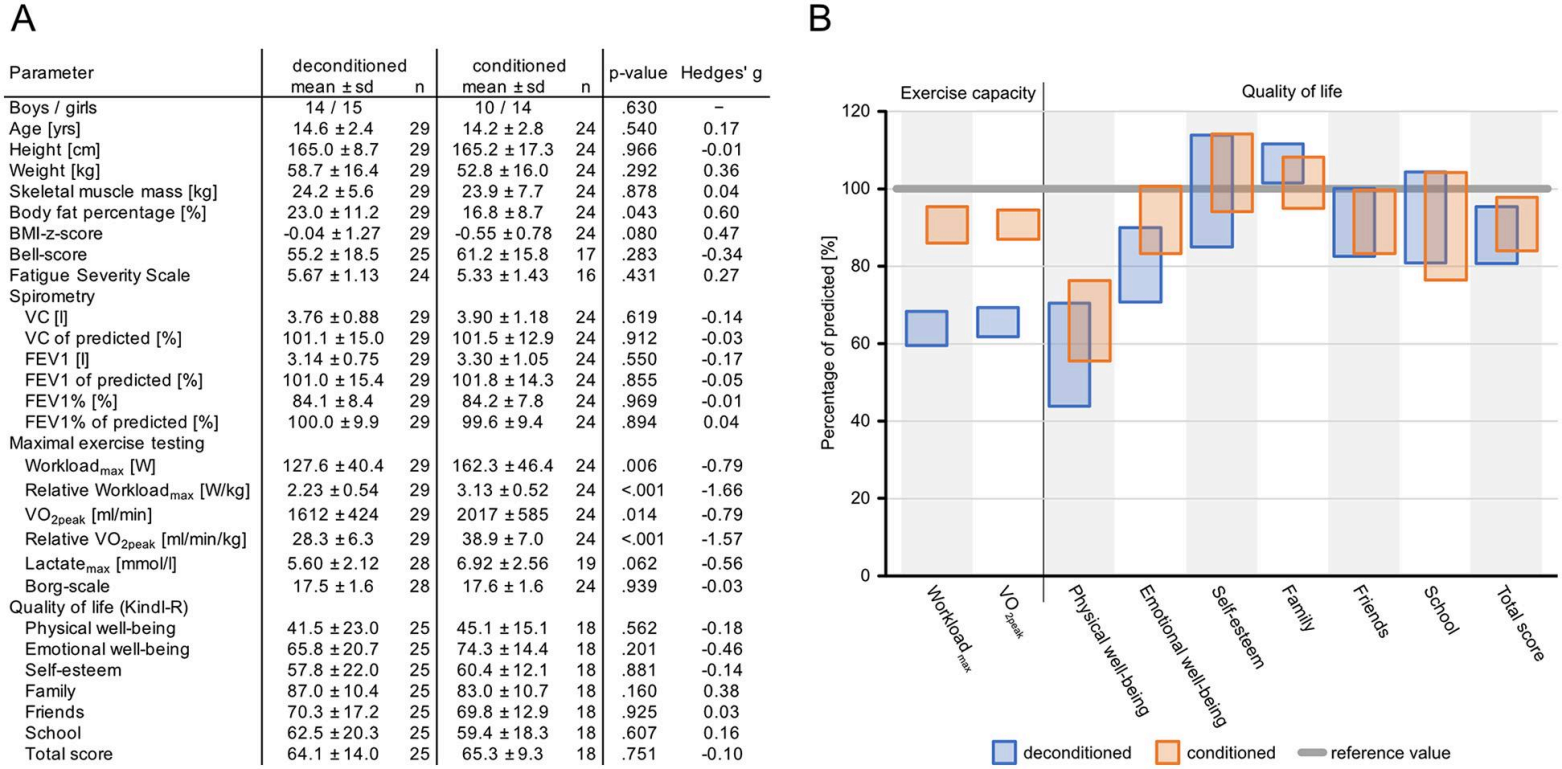
Cohort characteristics and clinical and wellbeing characteristics in conditioned vs. deconditioned pediatric patients with PCS. **(A)** Participant characteristics. **(B)** Exercise capacity and QoL expressed as a percentage of normative values. Data are shown as 95% confidence intervals.

To further assess to what extent reduced EC affects QoL in pediatric patients with PCS and which factors may have affected their physical activities, we grouped our cohort into conditioned patients with >80% of predicted VO_2peak_ during exercise testing vs. deconditioned patients with <80% VO_2peak_ in the absence of any signs of pulmonary or circulatory pathology. Figure 1A shows the presence of factors such as age, body weight, height, muscle mass, fat percentage, or BMI. In addition, analyses for differences in sex distribution, lung function testing, and the assessment of neuropsychological symptoms such as Bell Score and FSS yielded no significant differences between the two groups of patients. However, the elapsed time between infection and exercise testing differed significantly between the two groups (deconditioned vs. conditioned mean of 198.5 ± 120.7 vs. 230.8 ± 62.6 days; *p* = 0.021). In accordance with grouping, maximum workloads differed significantly, as did VO_2peak_ (Figure 1A), but lactate levels and self-assessed exertion did not differ between the groups (Figure 1A).

In QoL, as well as physical parameters in deconditioned vs. conditioned patients, there were no significant differences in any of the measured parameters (Figure 1A). In addition, values for self-esteem and QoL related to family, friends, and school were comparable between the groups. Accordingly, overall QoL did not differ between the two groups (Figure 1B).

## Discussion

Our results provide first-time data on comprehensively assessed exercise tolerance and QoL in pediatric patients with PCS and show a reduced EC in this patient group. These findings are similar to those of a recent study in adult patients with PCS (10). Exercise tolerance was particularly low in the deconditioned group, which displayed shorter times post SARS-CoV-2 infection, compared with that in the conditioned group. Furthermore, we observed no differences in wellbeing in pediatric patients with PCS having higher EC (conditioned state) compared with deconditioned patients, suggesting that neither a better training status before the infection nor a maintained EC after the infection prevents reductions in the self-perceived markers of physical and mental comfort. Our findings of reduced EC are in line with previous reports that showed limited EC in young PCS patients as analyzed by a self-assessment/questionnaire (6) or a 6 min walk test in limited cases (11). Importantly, however, we here applied the methodological gold standard in exercise testing and show an objective reduction in EC, which was not captured by self-assessment. With regard to QoL, pediatric patients with PCS reported slightly lower levels than normal, and QoL or self-esteem were not affected by exercise performance.

Our finding that longer time periods post SARS-CoV-2 infection are related to better EC levels is in accordance with observations by others who showed that PCS symptoms wane over time (2). Interestingly, lung function parameters such as VC, measurements such as lactate levels, or self-assessed EC did not affect exercise testing in our cohort. This varied among adult PCS patients, in whom VC was associated with exercise intolerance (4). In general, pediatric PCS differs significantly from adult PCS: Hallmark symptoms such as fatigue and abnormal lung function are reported less frequently in children than in adults (12, 13), and it has been speculated that this may be due to different immune responses, greater physiological resilience, fewer comorbidities, enhanced regenerative capacity, and potentially different viral dynamics. As pathophysiological mechanisms are unclear, management in children and adults mainly focuses on symptom relief, with moderate physical training as a possible component in interdisciplinary treatment approaches (14).

A limitation of our study is its monocentric nature and relatively small study population, which in part may also be due to the fact that PCS is less frequently diagnosed in children than in adults (15). A discussion on cardiac function monitoring and further pulmonary imaging would have been desirable and will be the objective in further research activities of our group. In addition, our study focused on Caucasian patients and may not be easily extrapolated on other populations or ethnicities.

In summary, we show that pediatric PCS is associated with reduced EC. This reduced fitness in PCS is associated with shorter times post SARS-CoV-2 infection, but this does not appear to affect the QoL or self-esteem of the patients in this study. We hope that our data will help in unraveling the pathophysiology of PCS across age groups to reveal novel diagnostic and therapeutic approaches to this condition.

## Data Availability

The raw data supporting the conclusions of this article will be made available by the authors without undue reservation.
